# Trends and forecast of drug-resistant tuberculosis: a global perspective from the GBD study 2021

**DOI:** 10.3389/fpubh.2025.1550199

**Published:** 2025-03-26

**Authors:** Yi Guo, Jiacheng Li, Lihong Lin

**Affiliations:** ^1^Department of Nosocomial Infection Control, The Quzhou Affiliated Hospital of Wenzhou Medical University, Quzhou People’s Hospital, Quzhou, China; ^2^Department of Vascular Surgery, The Quzhou Affiliated Hospital of Wenzhou Medical University, Quzhou People’s Hospital, Quzhou, China

**Keywords:** age-standardized incidence rate, estimated annual percentage change, prevention, drug-resistant tuberculosis, Global Burden of Disease

## Abstract

**Background:**

Tuberculosis (TB) is an infectious disease caused by *Mycobacterium tuberculosis*. Drug-resistant tuberculosis (DRTB) includes multidrug-resistant tuberculosis without extensive drug resistance (MDRTB) and extensively drug-resistant tuberculosis (EDRTB). Recently, with the continued rise of DRTB, global TB prevention and control efforts have faced even greater challenges.

**Aims:**

This study aimed to quantify the changes in age-standardized incidence rate (ASIR) of two types of DRTB from 1991 to 2021 using the Global Burden of Disease (GBD) database, and to examine the epidemiological differences across various regions and countries and applied the autoregressive integrated moving average (ARIMA) model to predict the epidemiological trends of MDRTB and EDRTB from 2022 to 2030.

**Methods:**

Data were extracted from the GBD database from 1991 to 2021. Estimated annual percentage changes (EAPC) in DRTB ASIR by regions, were calculated to quantify the temporal trends. ARIMA model was applied to predict ASIR between 2022 and 2030.

**Results:**

From 1991 to 2021, the global composition of DRTB shifted, with EDRTB increasing in developed regions and MDRTB remaining dominant in regions like sub-Saharan Africa. The highest ASIRs for MDRTB in 2021 were seen in Somalia, while the highest for EDRTB were in Moldova. Significant regional variations were observed, with East Asia showing a decrease in MDRTB and Oceania experiencing large increases in both MDRTB and EDRTB. Additionally, country-specific trends varied widely, with Slovenia showing the greatest decrease in MDRTB and Papua New Guinea the largest increase in EDRTB.

**Conclusion:**

This study highlights the ongoing dominance of MDRTB in low SDI regions and the expected decline of EDRTB in high SDI regions due to improved treatments and diagnostics. Global predictions suggest a reduction in DRTB burden by 2030, with a focus on early diagnosis and treatment optimization.

## Background

Tuberculosis (TB) is an infectious disease caused by *Mycobacterium tuberculosis*, primarily transmitted through airborne droplets, and mainly affects the lungs (1). Despite significant progress in TB treatment and control in recent years, it remains one of the major public health issues worldwide. Recent studies indicate that in 2019, there were approximately 10 million new TB cases globally, with 88% of them being adults. In the same year, TB led to approximately 1.2 million deaths (among HIV-negative individuals) (2). Beyond its health threats, TB also exacerbated issues such as poverty, malnutrition, and social discrimination, especially in resource-poor regions (3). With the rise of Drug-resistant tuberculosis (DRTB), the burden of TB disease has become even more complex (4). Given the distinct clinical progression (5), treatment response (6), and mortality outcomes (7) of DRTB in HIV-positive and HIV-negative individuals, it is crucial to analyze these two populations separately. According to the definitions of DRTB in the Global Burden of Disease (GBD) database, DRTB includes multidrug-resistant tuberculosis without extensive drug resistance (MDRTB) and extensively drug-resistant tuberculosis (EDRTB). The former is a form of TB (among HIV-negative individuals) that does not respond to the two most effective first-line antituberculosis drugs, but is not resistant to any fluoroquinolone and any second-line injectable drugs (8). And the latter is a form of TB (among HIV-negative individuals) which is not responsive to isoniazid, rifampicin, fluoroquinolones, and second-line injectable drugs (9). In recent years, with the continued rise of DRTB, global TB prevention and control efforts have faced even greater challenges. Among these, EDRTB became a key challenge due to its poor treatment outcomes, high costs, and the need for complex treatment regimens (10).

Although several studies have explored the epidemiological trends of DRTB, most focus on specific countries or regions and examine short-term analyses, lacking a systematic evaluation of long-term trends. Notably, the global epidemiological trends of DRTB vary significantly due to differences in regional development levels, health policies, and intervention measures. Additionally, the burden of MDRTB in low socio-demographic index (SDI) regions was much higher than in high SDI regions, and the lack of healthcare resources and limited diagnostic methods in these regions exacerbate the complexity of treatment and the risk of transmission (11). Moreover, the COVID-19 pandemic has significantly disrupted TB control programs, leading to delayed diagnoses, treatment interruptions, and increased transmission of DRTB (12). These challenges underscore the need for continued surveillance of DRTB trends in the post-pandemic era. Meanwhile, advancements in diagnostic technology, such as the introduction of GeneXpert MTB/RIF has revolutionized TB diagnosis, enabling rapid detection of *Mycobacterium tuberculosis* and rifampicin resistance within hours, compared to weeks with conventional methods (13), which significantly improved early MDRTB and EDRTB detection (14). Given these challenges and advancements, this study examines global MDRTB and EDRTB trends while incorporating the effects of COVID-19 and the impact of advanced diagnostics on disease burden estimates. By providing a systematic long-term assessment, this study highlights the necessity of sustained epidemiology monitoring to inform targeted public health interventions.

This study bases on the GBD database to comprehensively explore the global epidemiological trends of DRTB. The GBD database is currently one of the most comprehensive epidemiological databases, covering long-term trend data of multiple diseases in 204 countries and regions. It offers advantages such as rich indicators, wide coverage, and comprehensive analysis dimensions, providing scientific evidence for public health policies in different regions and countries (15). This database encompasses data on disease burden from 1990 to 2021, including metrics such as incidence, prevalence, mortality, and disability-adjusted life years (DALYs). Compared to prevalence and mortality, incidence offers unique value in assessing the effectiveness of health interventions, setting priorities for resource allocation, and formulating early prevention strategies. ASIR, defined as the number of new cases per 100,000 people after age adjustment (16), removes the confounding effect of age structure, providing a clearer reflection of disease risk, which is thus ideal for evaluating temporal trends of diseases at global or regional levels. The primary goal of this study was to quantify the changes in ASIR of two types of DRTB from 1991 to 2021 using the GBD database, and to examine the epidemiological differences across various regions and countries. Additionally, we used the autoregressive integrated moving average (ARIMA) model to predict the epidemiological trends of DRTB from 2022 to 2030. By revealing the long-term trends of DRTB, this study not only fills the existing gaps but also provides direction for optimizing regional intervention measures, thereby enabling more effective global health resource allocation, particularly in high-burden regions with scarce healthcare resources. Furthermore, this study contributes to the optimization of diagnostic technologies, improvement of treatment protocols, and precision in preventive and control measures. This has significant implications for achieving the End TB Strategy (17) and Sustainable Development in 2023 (18).

## Materials and methods

### Data source

All data in this study are available in Global Health Data Exchange (GHDx) online query tool,^1^ which is a comprehensive database that evaluates the global incidence, prevalence, years lived with disability (YLDs), DALYs, and healthy life expectancy (HALE) for 371 diseases and injuries across 204 countries and territories conducted by the Institute for Health Metrics and Evaluation (IHME) (19). In recent years, significant advancements in DRTB diagnostic technology, such as whole-genome sequencing and improved GBD methodologies, have enhanced data accuracy, although some degree of estimation bias remains. Fortunately, each release of the GBD recomputes the entire historical time series for a disease so that changes in case definitions, historical datasets and methods do not lead to spurious comparisons with past assessments (20). Annual incidence and ASIR with corresponding 95% uncertainty interval (UI) of MDRTB and EDRTB from 1991 to 2021, by regions, countries were extracted. These countries and territories are categorized into 5 regions by SDI, including low, low-middle, middle, high-middle, high SDI region, which are also separated into 21 regions in terms of geography.

### Data analysis

R Software (Version 4.4.1) with packages including ggplot2, ggsci, ggmap, sf, terra, maps, dplyr, forecast, tseries, aTSA was used for statistical analysis. All tests were two-sided, with a significance level of *α* = 0.05.

ASIR and estimated annual percentage change (EAPC) were used to quantify the incidence trends of MDRTB and EDRTB. ASIR is defined as the number of new cases per 100,000 people after age adjustment, which serves as a good surrogate for changing patterns of disease within a population, as well as clues to the changing risk factors (16). EAPC is a summary and a widely used measurement of the ASIR trend over a specified interval (from 1991 to 2021), of which the regression model is fitted as follow, and EAPC’s corresponding confidential interval (CI) can also be obtained from the regression model (21):


$$\ln\left(\mathrm{ASIR}\right)=\alpha +\beta *\mathrm{calendar year}+\varepsilon$$

$$\mathrm{EAPC}=100*\left(e^{\beta -1}\right)$$

First, percent bar charts were used to described the composition of incident cases of DRTB in 1991 and 2021 globally and across regions. Then, the distributions of ASIR in 2021 and ASIR EAPC of MDRTB and EDRTB were described through statistic map across 204 countries and territories. A bar chart was also used to describe the EAPCs and their 95% CI of MDRTB and EDRTB globally and across regions. Additionally, the association between EAPCs and ASIRs (1991) at national level was assessed. Finally, we used ARIMA model to forecast ASIR of MDRTB and EDRTB between 2022 and 2030. The auto.arima() function was employed to automatically select the autoregression order (AR(*p* = 0)), moving average order (MA(q = 0)), and the degree of difference (I(d = 2)) of the ARIMA model. This function optimizes information criteria (e.g., AIC or BIC) to search for the best model parameter combination, thereby avoiding potential biases from subjective selection. The ARIMA model was constructed based on ASIR data reported from 1991 to 2021. Since the concept of DRTB was standardized by WHO in Guidelines for the programmatic management of drug-resistant tuberculosis published in 2008, sensitivity analysis of the ARIMA model was performed by excluding data from 1991 to 2008. This forecasting allows us to better comprehend potential trends and plan interventions accordingly.

## Results

### Changes in compositions of incident cases of DRTB from 1991 to 2021

**Figure 1:**
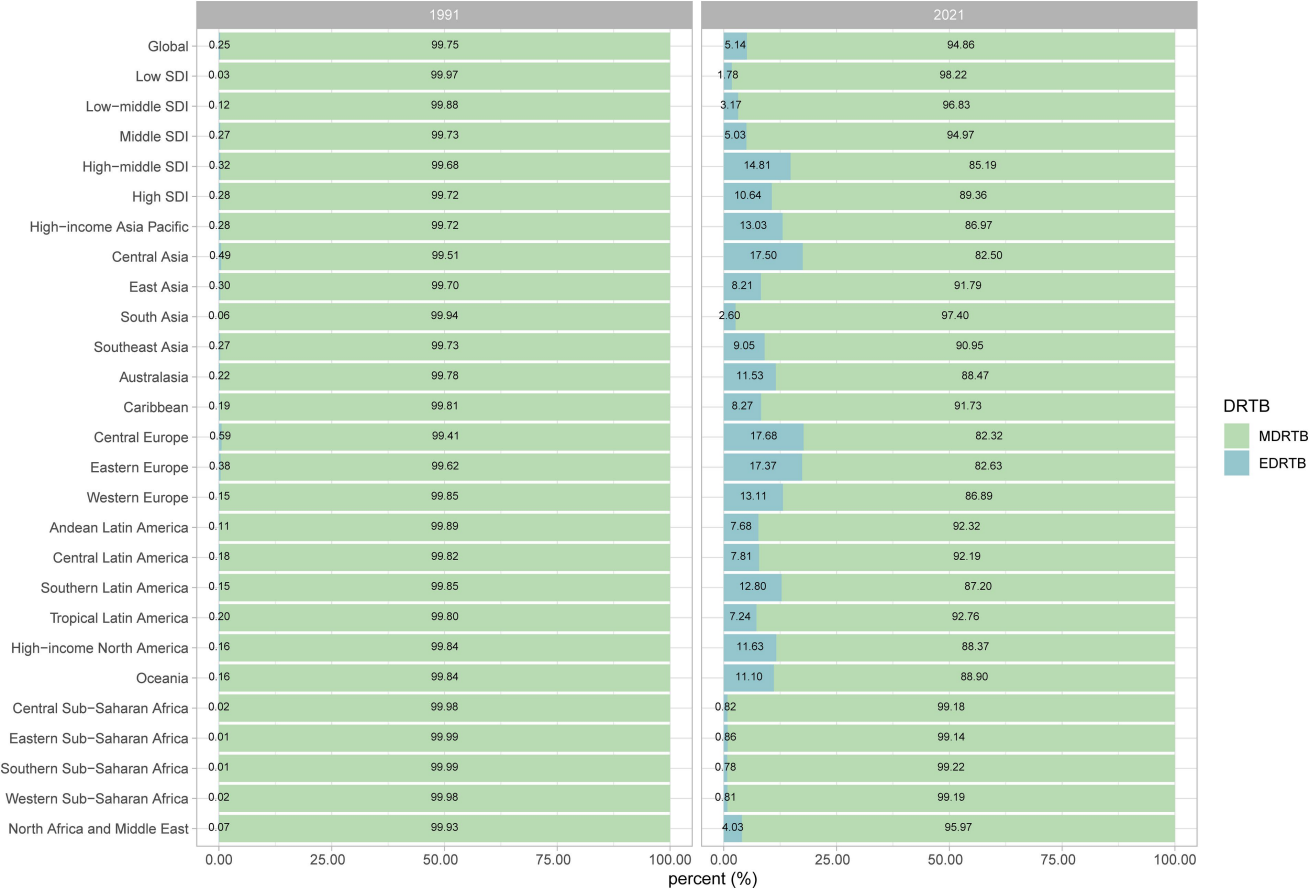
Compositions of the incident cases of DRTB at regional level.

From 1991 to 2021, the composition of incident cases of DRTB underwent varying degrees of changes globally and across regions. In 1991, the proportion of EDRTB was extremely low globally and in all regions (all below 1%), with MDRTB accounting for nearly all DRTB incident cases. By 2021, the proportion of EDRTB had significantly increased both globally and regionally, with developed regions such as central Asia, central Europe, eastern Europe, and high-income north America exhibiting higher proportions of EDRTB compared to other regions. Central Europe showed the most pronounced increase, with the proportion of EDRTB rising from an initial 0.59% to 17.68%. In contrast, while the proportion of EDRTB cases in sub-Saharan African regions also increased to varying degrees, MDRTB continued to dominate the composition of DRTB cases, consistently accounting for over 99% (Figure 1).

### Global DRTB burden

**Figure 2:**
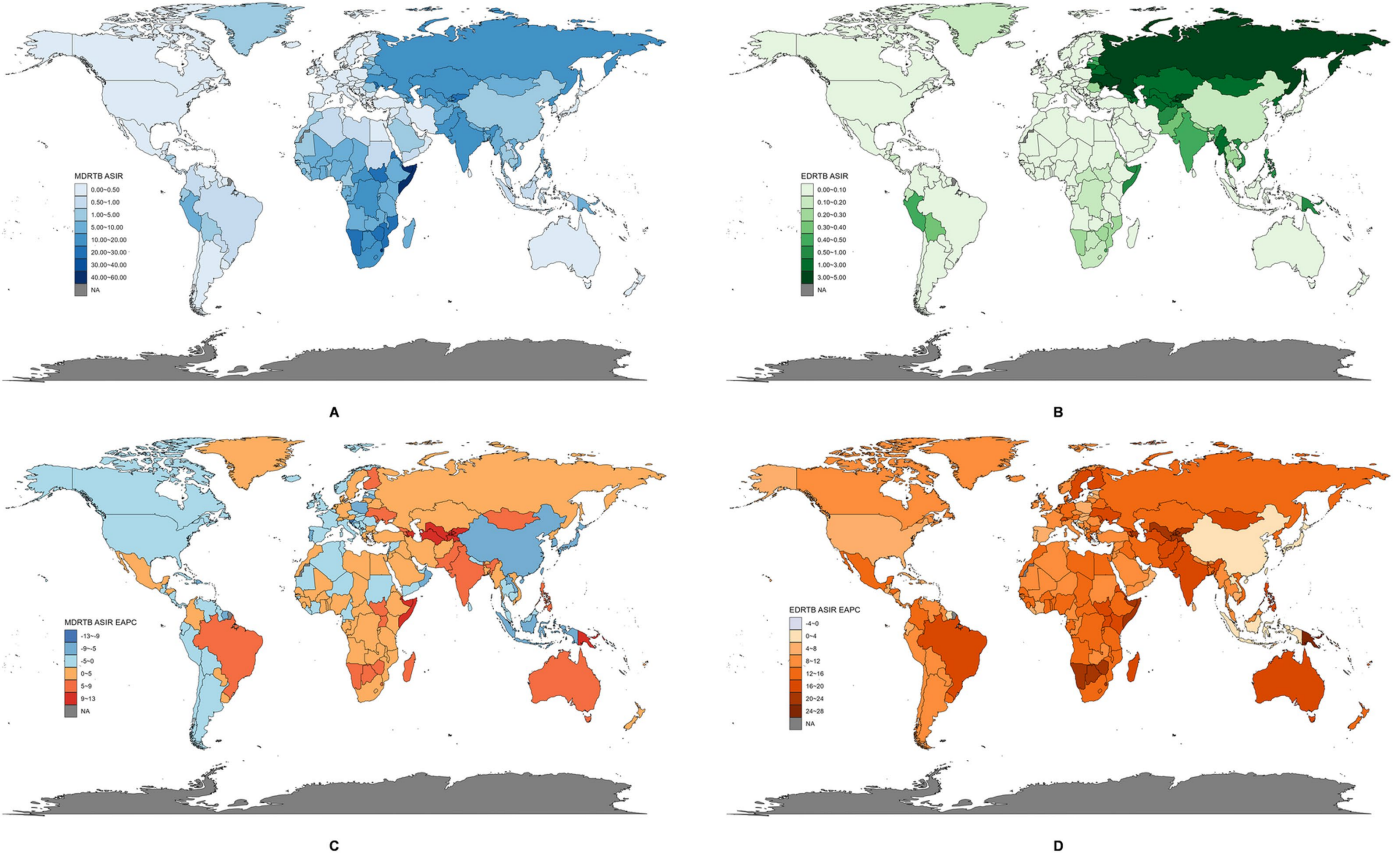
The global disease burden of DRTB in 204 countries and territories. **(A)** ASIR of MDRTB in 2021. **(B)** ASIR of EDRTB in 2021. **(C)** EAPC of MDRTB between 1991 and 2021. **(D)** EAPC of EDRTB between 1991 and 2021.

The ASIR of DRTB varied considerably across the world. For MDRTB’s ASIR in 2021 (Figure 2A), the highest was observed in Somalia (57.25, 95% UI: 14.12~169.56) and the lowest in Slovenia (0.004, 95% UI: 0.001~0.014). For EDRTB’s ASIR in 2021 (Figure 2B), the highest was observed in Republic of Moldova (4.43, 95% UI: 1.41~8.44), and the lowest in Slovenia (0.0008, 95% UI: 0.0001~0.0029).

There were also large differences among EPAC of ASIR of DRTB across countries and territories. For MDRTB (Figure 2C), the greatest decrease was observed in Slovenia (−12.59, 95% CI: −14.11 to −11.05), and the highest increase in Kyrgyzstan (12.80, 95% CI: 9.65~16.04). For EDRTB (Figure 2D), the greatest decrease was observed in Barbados (−2.21, 95% CI: −4.35 to −0.02), and the highest increase in Papua New Guinea (24.36, 95% CI: 20.23~28.63).

**Figure 3:**
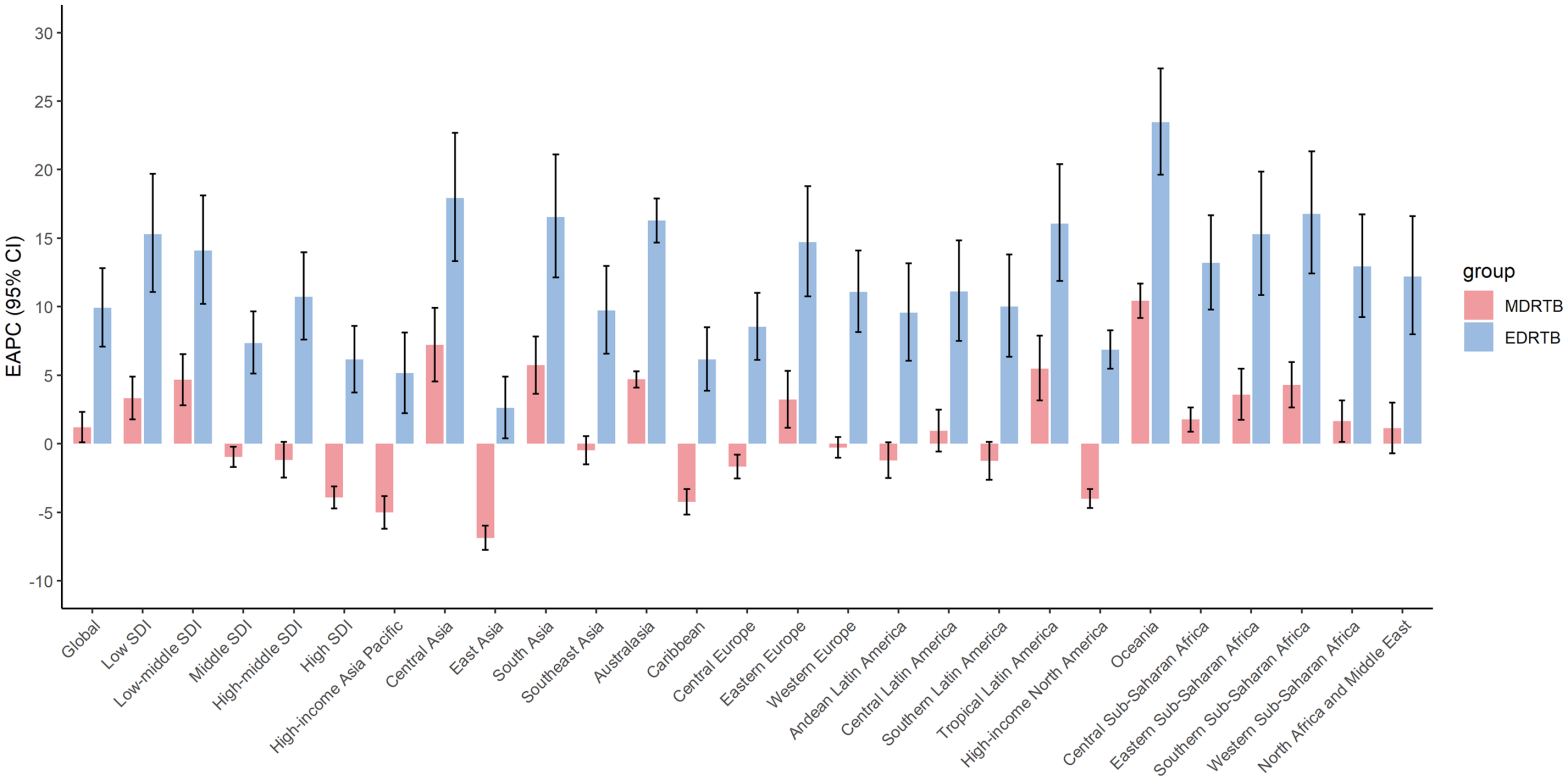
EPACs of DRTB ASIR from 1991 to 2021.

Figure 3 showed EPACs and their 95% CI globally and by regional levels. For MDRTB, ASIR increased significantly globally (1.20, 95% CI: 0.10~2.31); it was deemed to be stable in regions like high-middle SDI region, southeast Asia, western Europe, central Latin America, etc.; the most significant decrease was observed in East Asia (−6.86, 95% CI: −7.75 to −5.96) and the most significant increase was observed in Oceania (10.43, 95% CI: 9.19~11.70). For EDRTB. ASIR also increased significantly globally (9.91, 95% CI: 7.10~12.81) and in all regions; the highest increase was observed in Oceania (23.45, 95% CI: 19.64~27.39) and the lowest increase was observed in East Asia (2.61, 95% CI: 0.39~4.89).

**Figure 4:**
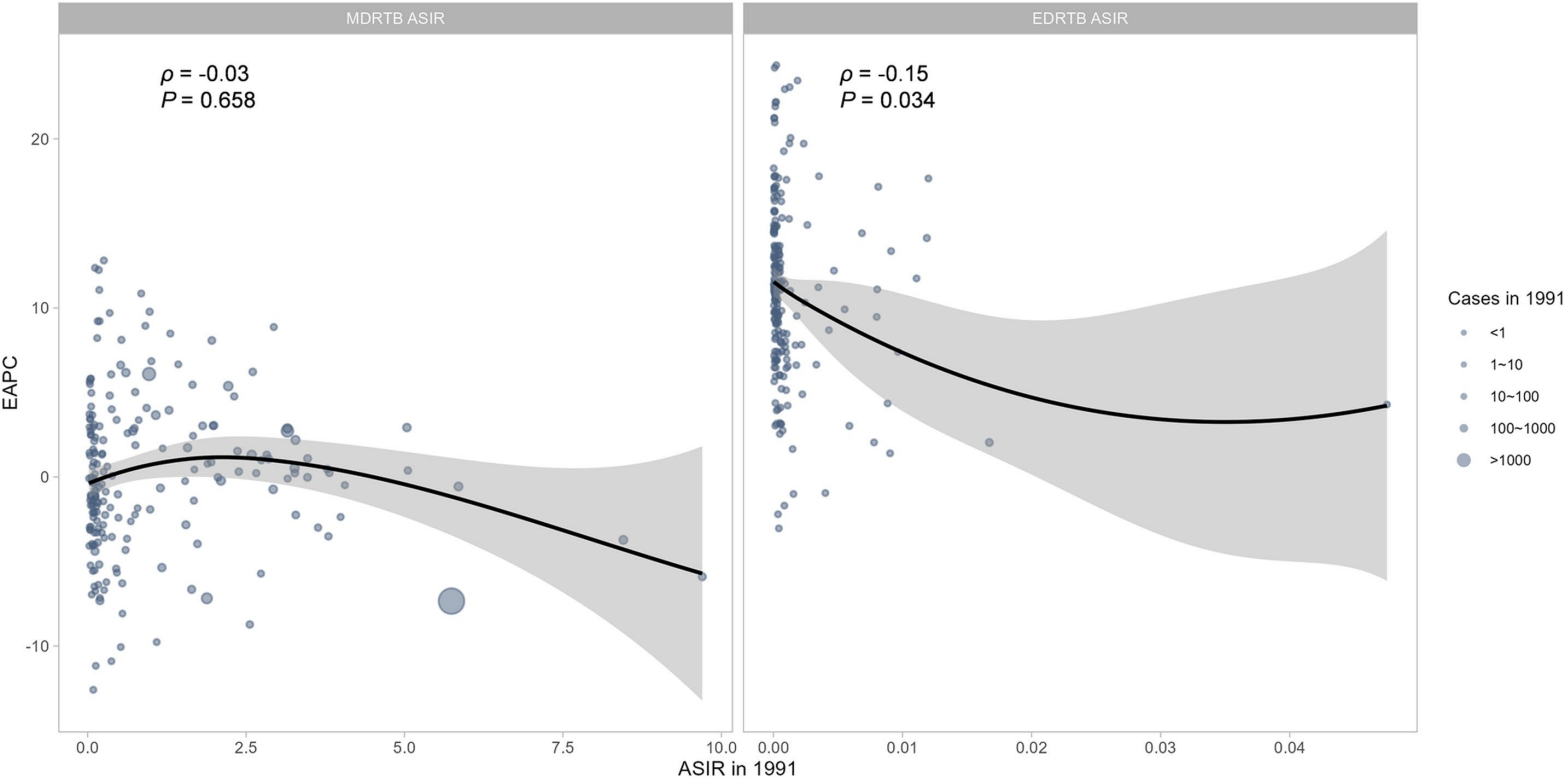
The correlation between EAPC and ASIR in 1991.

The ASIRs of DRTB in 1991 reflects the baseline disease burden. As shown in Figure 4, for MDRTB, there was no significant correlation between EPAC and ASIR in 1991, but there might exist a negative association when the ASIR was above 2.5. And a significant negative correlation was found in EDRTB ASIR in 1991 and EAPC (*ρ* = −0.15, *p* = 0.034).

### Forecast analysis for ASIR of DRTB

**Figure 5:**
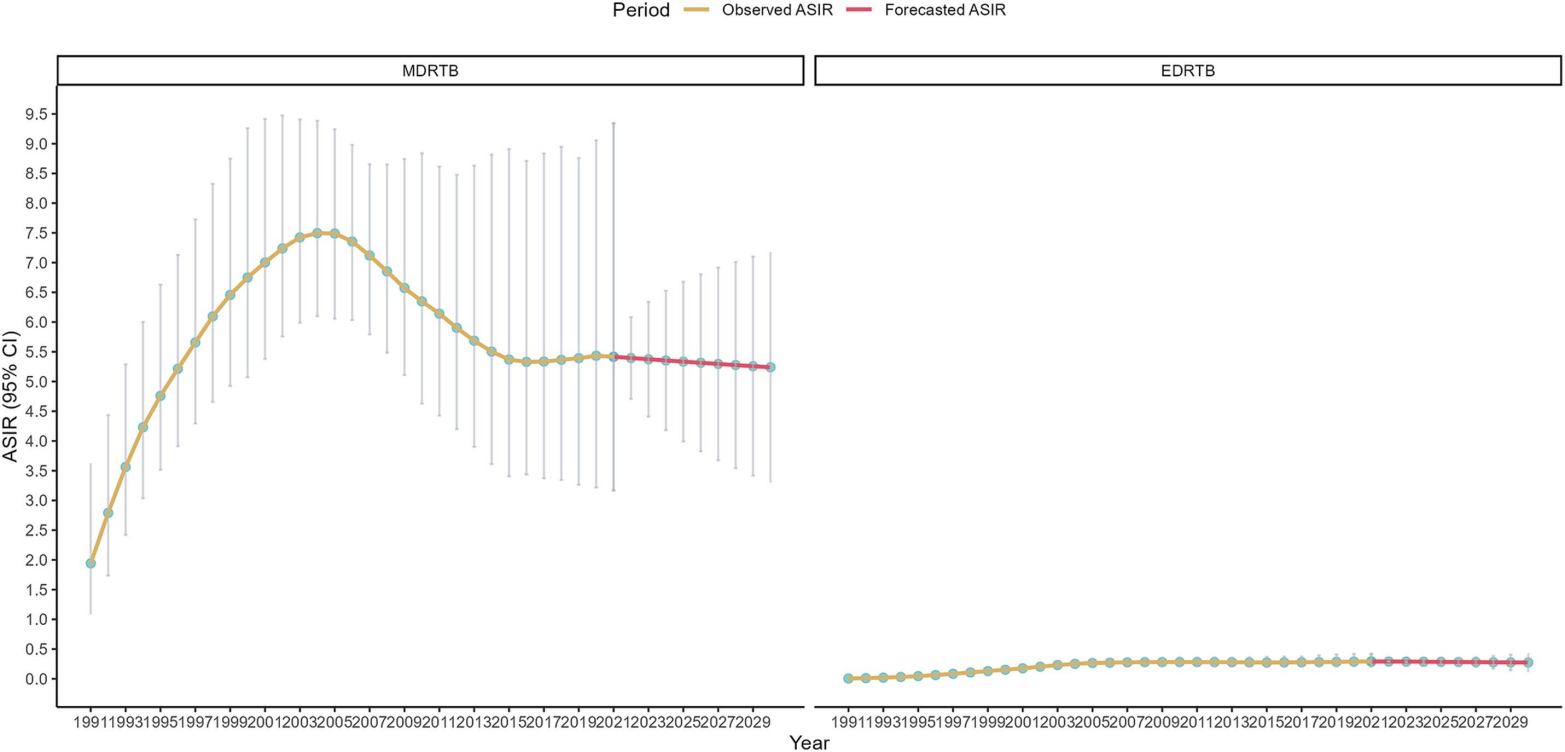
ASIR of DRTB assessed from 1990 to 2021 alongside forecasted values for the period between 2022 and 2030.

Results of the ARIMA model were shown in Figure 5. The study forecasted that the ASIR of MDRTB would be 5.40 (95% CI: 4.71~6.08) in 2022 and would decrease to 5.24 (95% CI: 3.30~7.18) in 2030. Additionally, ASIR of EDRTB would be 0.29 (95% CI: 0.28~0.30) in 2022 and would decrease to 0.27 (95% CI: 0.11~0.43).

**Figure 6:**
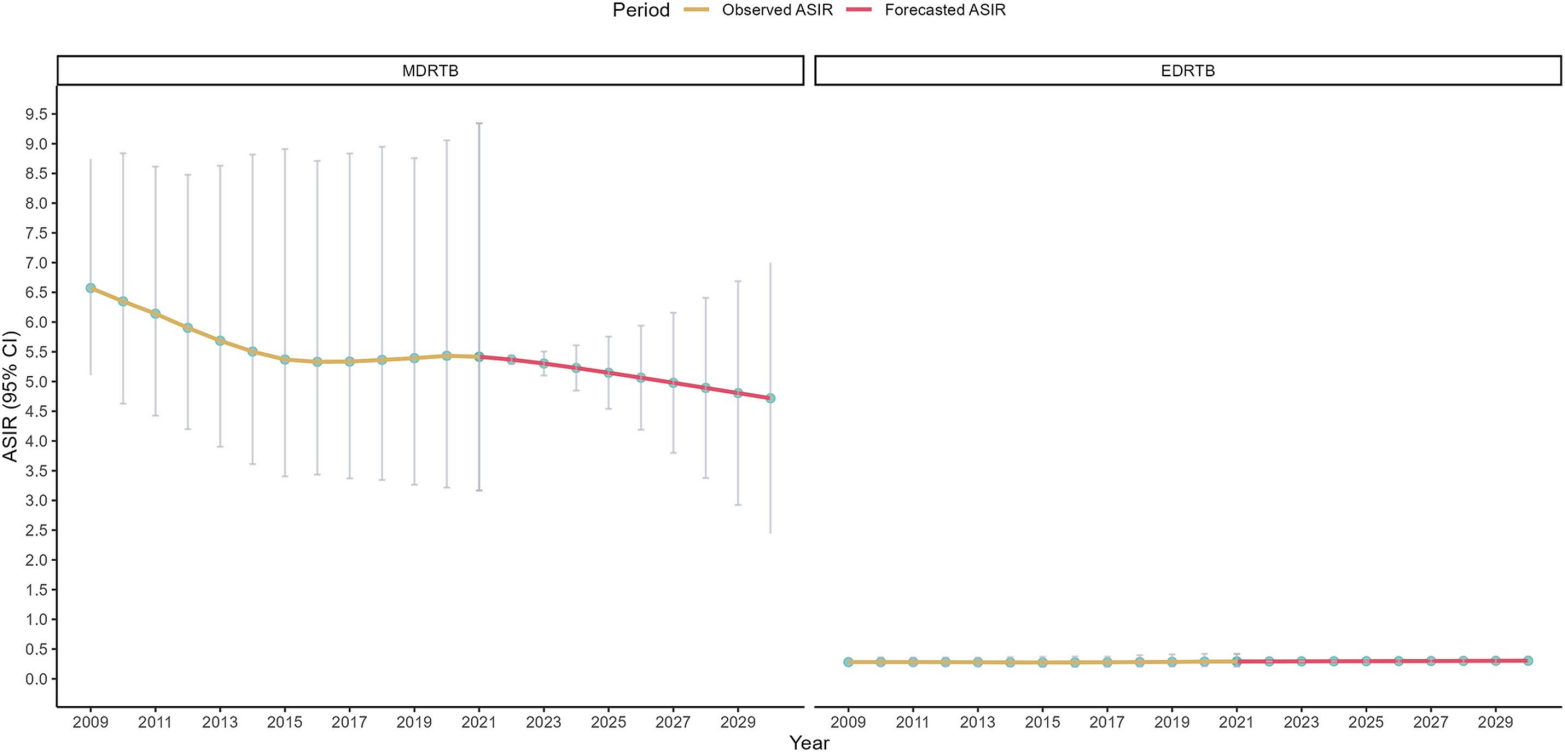
Sensitivity analysis results of ARIMA model.

Sensitivity analysis of ARIMA model were shown in Figure 6, which were not changed significantly compared to the main analysis. The study forecasted that the ASIR of MDRTB would be 5.37 (95% CI: 5.30~5.44) in 2022 and would decrease to 4.72 (95% CI: 2.44~6.99) in 2030. Additionally, ASIR of EDRTB would be 0.29 (95% CI: 0.29~0.30) in 2022 and would decrease to 0.30 (95% CI: 0.25~0.36).

## Discussion

Previous studies have analyzed MDRTB burden using GBD dataset, yet important gaps remain. Song et al. conducted a 30-year analysis of MDRTB burden based on GBD 2019, highlighting temporal trends, age-period-cohort effects, and key risk factors such as alcohol consumption (11). Similarly, Lv et al. (22) assessed MDRTB burden from 1990 to 2019, focusing on gender, age, and regional disparities. However, both studies primarily examined MDRTB and did not separately analyze EDRTB, despite its growing global burden. Moreover, neither study incorporated predictive modeling using real-world surveillance data to anticipate future MDRTB and EDRTB trends. Furthermore, the impact of the COVID-19 pandemic on DRTB epidemiology was not extensively explored in these studies, despite its potential to disrupt TB control programs globally. This study aims to address these gaps by providing a comprehensive analysis of DRTB trends from 1991 to 2021, with a specific focus on regional differences and long-term epidemiological patterns. Furthermore, by forecasting trends from 2022 to 2030, this study offers a reference for optimizing intervention measures. The strengths of this study lie first in the use of the authoritative and comprehensive epidemiological data from the GBD database; second, incorporating data from the COVID-19 pandemic period to assess its impact on DRTB burden and adopting indicators such as ASIR and EAPC; third, integrating the ARIMA model to ensure both scientific rigor and accurate future trend predictions (23).

The study revealed the global epidemiological trends and regional differences of DRTB. Our fundings align with the previous, which demonstrated that MDRTB incidence and DALYs were highest in South Asia and Sub-Saharan Africa, regions with significant healthcare disparities and limited access to second-line treatment (22). The regional variation in MDRTB and EDRTB incidence can be attributed to multiple factors, including improvements in diagnosis, adherence to treatment, policy interventions, and recent global health crises such as the COVID-19 pandemic. First, the rise in reported EDRTB cases in high-income regions may be partially explained by better access to molecular diagnostic tools (e.g., Xpert MTB/RIF, whole-genome sequencing), allowing for earlier and more accurate detection. Studies have shown that rapid molecular diagnostics significantly improve DRTB detection and treatment outcomes, particularly in well-resourced settings (24). Second, in low-SDI regions, MDRTB continues to dominate due to inconsistent treatment adherence, inadequate follow-up, and lack of access to second-line drugs (25). Poor adherence to treatment, often due to high medication costs, long treatment durations, and lack of patient support, contributes to the persistence of MDRTB. In contrast, high-SDI regions have better patient management systems and directly observed treatment programs, which may help contain MDRTB but also select for more EDRTB strains over time (26). Third, policy-driven factors also play a key role in regional differences. Countries with substantial healthcare investments and strong national TB control programs have reported declining MDRTB but rising EDRTB, likely due to better surveillance and case detection (27). Meanwhile, countries with low healthcare budgets and limited TB control resources (e.g., Sub-Saharan Africa, parts of South Asia) struggle with MDRTB containment, leading to persistent transmission in these settings (28). Additionally, international funding and technology transfer have helped certain middle-income countries reduce their MDRTB burden, although the sustainability of these efforts remains uncertain. Fourth, the COVID-19 pandemic significantly disrupted global TB control efforts, affecting diagnosis, treatment, and case reporting. The pandemic led to an 18% drop in TB case notifications in 2020, largely due to healthcare system strain and the diversion of diagnostic resources to COVID-19 testing (12). This likely resulted in underdiagnosis of MDRTB and EDRTB, particularly in resource-limited settings where access to molecular diagnostics was already limited. And many countries experienced a decline in reported MDRTB and EDRTB cases during 2020–2021, likely due to reduced surveillance rather than a true epidemiological decline (29). Although the GBD 2021 estimates adjust for these trends, caution is needed when interpreting short-term fluctuations.

These findings have significant clinical implications. First, regions with high EDRTB rates should enhance diagnostic capabilities and optimize treatment protocols to better manage DRTB-related challenges. For low SDI regions where MDRTB remained predominant, strengthening resistance detection and drug susceptibility testing was crucial for improving treatment success rates and reducing the incidence of DRTB (30). Additionally, as global trends of DRTB evolved, particularly in low SDI regions, there was an urgent need for more effective public health strategies and interventions. These included widespread drug resistance monitoring, promoting diagnostic technologies for early detection, optimizing treatment protocols, and improving adherence to tuberculosis treatment (31, 32). Particularly in resource-limited regions, enhancing the tracking and early screening of high-risk populations was critical.

The study also found a negative correlation between the EAPC of MDRTB and its baseline ASIR, indicating that although regions with high baseline ASIR had a heavier burden, their subsequent growth rate was relatively slow. Regions with high baseline ASIR typically had more complex control and prevention contexts. These regions may have already entered a relatively “stable” state: the prevalence of MDRTB placed significant pressure on their healthcare systems, making treatment and drug management more difficult. Despite improvements in early diagnostic capabilities, the rapid development of resistant strains and the complexity of treatment protocols prevented a significant reduction in the burden (33). Moreover, public health measures in high MDRTB burden regions often had a delayed effect. Although new technologies and diagnostic methods were gradually promoted, their impact was slow to materialize due to insufficient resource support. Therefore, in regions with heavy MDRTB burdens and lower EAPCs, more precise and targeted public health strategies were needed. Especially in regions with high baseline ASIR, early diagnosis of DRTB should be strengthened, treatment protocols optimized, and adherence to treatment improved to prevent further spread of resistant strains. For low-income regions, optimizing resource allocation, monitoring DRTB, and integrating data would be key areas for future prevention and control efforts. Public health policies needed to place more emphasis on infrastructure development, particularly in the areas of drug management and the widespread provision of tuberculosis control services. Additionally, the key to reducing MDRTB transmission in high-burden areas was enhancing the diagnostic capacity of primary healthcare institutions and improving access to treatment resources. This would help drive further improvement in EAPC and ultimately reduce the global burden of MDRTB (34).

Finally, the study used the ARIMA model to predict the overall trend of DRTB burden from 2022 to 2030. Globally, the burden of both types of DRTB would decrease in 2030. This change reflected the gradual strengthening of global tuberculosis control measures, particularly improvements in early diagnosis of DRTB, optimization of treatment protocols, and drug management. In high-income regions, the reduction in EDRTB burden was likely due to optimized treatment protocols and the widespread application of molecular diagnostic technologies, with the resources and technical support in these regions providing strong safeguards for controlling EDRTB (35). Meanwhile, low-income regions, despite facing resource scarcity, gradually improved treatment outcomes through international aid and technology transfer, which led to a reduction in the DRTB burden (36). It’s important to note that the definition of EDRTB changed in 2024, which emphasize a broader classification to bedaquiline and linezolid, reflecting shifts in clinical treatment strategies and evolving landscape of drug resistance (37). Given this shift, the predicted trends of EDRTB for 2022–2030 should be interpreted with caution, as the GBD dataset may not fully capture the epidemiological impact of these changes. Future public health strategies should focus on several key areas. First, the monitoring and data collection of DRTB should be strengthened. Public health institutions worldwide, especially in low-income countries, should enhance the monitoring of DRTB and timely collect and analyze resistance data (38). Second, the drug management system should be optimized. The control of MDRTB largely depends on the standardization of drug management. In low SDI regions, strengthening drug supply chain management is essential to ensure the timely availability and proper use of anti-tuberculosis drugs (39). Moreover, the promotion of more efficient drug combinations and short-term therapies is necessary to reduce the development of resistance. Third, improving treatment adherence is crucial. Enhancing patient adherence to treatment is key to controlling MDRTB, especially in low-income areas. Using digital adherence monitoring technologies and other innovative methods can help improve patient compliance, supported by telemedicine and patient management platforms (38). Lastly, international collaboration and resource sharing should be expanded. The control of DRTB relies not only on the efforts of individual countries or regions but also on global cooperation and resource sharing. Support from high-income countries to low-income countries will promote the synergistic effect of global anti-tuberculosis efforts. By sharing diagnostic technologies, treatment protocols, and data, the capacity for tuberculosis control in low-income countries can be accelerated (36).

This study has the following limitations. First, the burden analysis of EDRTB and MDRTB in this study mainly relied on ASIR, which, although accurately reflecting global epidemiological trends, did not fully account for the differences in healthcare resources across regions or the effects of treatment and adherence under varying socio-economic conditions. Second, although this study included data from 204 countries and regions in the GBD database, the interventions for tuberculosis control vary significantly across countries and regions. The study did not analyze in-depth how specific changes in diagnostic definitions, reporting accuracy, control measures and resource allocation in different regions affect the trends of DRTB. And data limitations remain a key challenge in interpreting DRTB trends, particularly in low-resource settings such as Somalia and Papua New Guinea, which was similar to previous studies (11, 22). Therefore, the results may not fully reflect the actual impact of public health interventions. The COVID-19 pandemic introduced additional uncertainties in DRTB estimates due to underreporting and diagnostic delays, as the ARIMA model depends on the accuracy of historical data. Given that the transmission of DRTB is influenced by various factors such as global tuberculosis control policies, drug supply, and socio-economic factors, the ARIMA model’s predictions may not fully capture the changes in these complex factors, especially the impact of unexpected public health events (such as the COVID-19 pandemic) on tuberculosis control. Finally, the definitional shift of EDRTB introduces potential biases in estimating long-term trends. Consequently, the ARIMA-based predictions for EDRTB from 2022 to 2030 should be interpreted cautiously, especially in high-income regions where newer drugs are widely used.

## Conclusion

This study conducted a comprehensive analysis of the global epidemiological trends of DRTB (including MDRTB and EDRTB) based on the GBD database. The results indicated that MDRTB continues to dominate in low SDI regions, with no significant reduction in the burden of MDRTB in low-income regions over time. In high SDI regions, although the burden of EDRTB has increased, it is expected that the burden of EDRTB will decrease in the coming years due to the optimization of treatment protocols and the application of molecular diagnostic technologies. Predictions from the ARIMA model suggest that the global burden of DRTB will generally decline between 2022 and 2030, with a particular reduction in the burden of EDRTB in high-income regions, while the burden of MDRTB in low-income regions may also be gradually controlled. These findings provide a scientific basis for the development of public health policies. Specifically, regions with high burdens should strengthen early diagnosis of DRTB, optimize treatment protocols, and increase the prevalence of drug susceptibility testing. Meanwhile, the control of MDRTB in low-income regions should continue to focus on drug management and treatment adherence to reduce the transmission of DRTB.

Further research should explore how specific public health interventions in different regions influence the trends of DRTB, particularly localized interventions in low-income and resource-limited regions and should evaluate the long-term effects of COVID-19 on DRTB trends, particularly whether diagnostic disruptions and treatment interruptions have led to an underestimation of the true burden. Additionally, assessing post-pandemic case rebound and its impact on drug resistance evolution is critical for future TB control strategies. Moreover, as HIV co-infection significantly affects TB outcomes, future studies should stratify DRTB burden by HIV status. Second, with the emergence of new drug-resistant tuberculosis strains, future research should focus on how genomic analysis and dynamic resistance monitoring can be used to predict and address the challenges posed by these new drug-resistant strains. Additionally, this study primarily relied on the GBD database and the ARIMA model for data analysis and trend prediction. Although these methods are widely used in epidemiological research, they still have limitations, such as data lag, assumptions and definitional shifts of diseases. Future research should integrate real-world clinical data and updated surveillance reports to validate long-term epidemiological forecasts. Additionally, subsequent GBD studies should explore alternative modeling approaches that account for definitional shifts and drug resistance evolution. Lastly, with the strengthening of global health cooperation, future research should also focus on how international collaboration and technology transfer can enhance tuberculosis control capabilities in low-income countries and reduce the global burden of MDRTB and EDRTB. The optimization of global health resources and cross-border data sharing will be key areas of focus for future public health efforts.

## Data Availability

The data underlying this article are available in Global Burden of Diseases, at https://vizhub.healthdata.org/gbd-results/ and further inquries can be directed to the corresponding author.

## Footnotes

^1^ https://vizhub.healthdata.org/gbd-results/
